# Supplementary material for: Facing the Human and Animal Brucellosis Conundrums: The Forgotten Lessons
Source: Microorganisms. 2022 Apr 30;10(5):942. doi: 10.3390/microorganisms10050942 (PMC9144488; doi:10.3390/microorganisms10050942)
Supplement: Supplementary file 1 [file microorganisms-10-00942-s001.zip › microorganisms-1593010-supplementary.pdf]

**Supplemental material. PCR studies**

A PubMed search was performed querying for ((((((((Brucellosis[Title/Abstract])) OR (Brucella[Title/Abstract])) AND (PCR[Title/Abstract]))) ) AND (diagnosis[Title/Abstract])) NOT (human[Title/Abstract]) identifies 169 works until 2021/12/27. Then, the authors scanned abstracts and titles and selected works specifically dealing with (1) amplification methods aimed to detect *Brucella* (tested *in vitro*) and (2) direct diagnosis of S *Brucella* and *B. ovis* in domestic livestock

**1. Only** amplification **methods (no diagnostics) with/without analytical Se/Sp (typing and vaccine identification excluded)**

**Before 2000**


1. Baily GG, Krahn JB, Drasar BS, Stoker NG. 1992. Detection of *Brucella melitensis* and *Brucella abortus* by DNA amplification. J Trop Med Hyg 95:271–275.
2. Herman L, De Ridder H. 1992. Identification of *Brucella* spp. by using the polymerase chain reaction. Appl Environ Microbiol 58:2099–2101.
3. Ouahrani-Bettach S, Soubrier MP, Liautard JP 1996.1S6501- anchored PCR for the detection and identification of *Brucella* species and strains. J. Appl. Biotechnol. 81: 154-160
4. Redkar et al., 2001. Real-time detection of *Brucella abortus*, *Brucella melitensis* and *Brucella suis*. Molecular and Cellular Probes 2001 15, 43–52
5. Newby DT, Hadfield TL, Roberto FF. 2003. Real-Time PCR Detection of *Brucella abortus*: a Comparative Study of SYBR Green I, 5′-Exonuclease, and Hybridization Probe Assays. Appl Environ Microbiol 69:4753–4759.
6. Probert WS, Schrader KN, Khuong NY, Bystrom SL, Graves MH. 2004. Real-time multiplex PCR assay for detection of *Brucella* spp., *B. abortus, and B. melitensis.* J Clin Microbiol 42:1290–1293.
7. Hinić V, Brodard I, Thomann A, Holub M, Miserez R, Abril C. 2009. IS711-based real-time PCR assay as a tool for detection of Brucella spp. in wild boars and comparison with bacterial isolation and serology. BMC Vet Res 5:1–8.
8. Bounaadja L, Albert D, Chénais B, Hénault S, Zygmunt MS, Poliak S, Garin-Bastuji B. 2009. Real-time PCR for identification of Brucella spp.: A comparative study of IS711, bcsp31 and per target genes. Vet Microbiol 137:156–164.
9. Kumar S, Tuteja U, Sarika K, Singh D, Kumar A, Kumar O. 2011. Rapid multiplex PCR assay for the simultaneous detection of the Brucella Genus, B. abortus, B. melitensis, and B. suis. J Microbiol Biotechnol 21:89–92.
10. Mirnejad R, Doust RH, Kachuei R, Mortazavi SM, Khoobdel M, Ahamadi A. 2012. Simultaneous detection and differentiates of *Brucella abortus* and *Brucella melitensis* by combinatorial PCR. Asian Pac J Trop Med 5:24–28.
11. Matero P, Hemmila H, Tomaso H, Piiparinen H, Rantakokko-Jalava K, Nuotio L, Nikkari S. 2011. Rapid field detection assays for Bacillus anthracis, *Brucella* spp., *Francisella tularensis* and *Yersinia pestis*. Clin. Microbio. lInfect 17:34–43.
12. Selim AM, Elhaig MM, Gaede W. 2014. Development of multiplex real-time PCR assay for the detection of *Brucella* spp., *Leptospira* spp. and Campylobacter foetus. Vet Ital 50:269–275.
13. Qasem JA, AlMomin S, Al-Mouqati SA, Kumar V. 2015. Characterization and evaluation of an arbitrary primed Polymerase Chain Reaction PCR product for the specific detection of *Brucella* species. Saudi J Biol Sci 22:220–226.
14. Sung S-R, Erdenebaataar J, Vanaabaatar B, Jung SC, Park YH, Yoo H-S, Her M. 2016. Differential diagnosis of *Brucella abortus* by real-time PCR based on a single-nucleotide polymorphisms. J Vet Med Sci 78:557–562.
15. Prusty BR, Chaudhuri P, Chaturvedi VK, Saini M, Mishra BP, Gupta PK. 2016. Visual Detection of *Brucella* spp. in Spiked Bovine Semen Using Loop-Mediated Isothermal Amplification LAMP Assay. Indian J Microbiol 56:142–147.

16. Hull N, Miller J, Berry D, Laegreid W, Smith A, Klinghagen C, Schumaker B. 2018. Optimization of *Brucella abortus* Protocols for Downstream Molecular Applications. J Clin Microbiol 56.

17. Dao TNT, Lee EY, Koo B, Jin CE, Lee TY, Shin Y. 2018. A microfluidic enrichment platform with a recombinase polymerase amplification sensor for pathogen diagnosis. Anal Biochem 544:87–92.

18. Sebastiani et al. 2018. A multi-screening Fast qPCR approach to the identification of abortive agents in ruminants. J Microbiol Methods 148:12–17.

19. Boby N, Ali SA, Preena P, Kaur G, Kumar S, Chaudhuri P. 2018. Detection of multiple organisms based on the distance-dependent optical properties of gold nanoparticle and dark-field microscopy. Talanta 188:325–331.

20. Bhat IA, Mashooq M, Kumar D, Varshney R, Rathore R. 2019. Development of probe-based real-time loop-mediated isothermal amplification for detection of *Brucella*. J Appl Microbiol 126:1332–1339.

21. Gumaa MM, Cao X, Li Z, Lou Z, Zhang N, Zhang Z, Zhou J, Fu B. 2019. Establishment of a recombinase polymerase amplification (RPA) assay for the detection of *Brucella* spp. Infection. Mol Cell Probes 47:101434.

22. Mascarenhas et al. 2020. Validation of real-time PCR technique for detection of *Mycobacterium bovis* and *Brucella abortus* in bovine raw milk. Brazilian J Microbiol. 51:2095–2100.

23. Nosaz et al. 2020. Development of a DNA aptamer to detect *Brucella abortus* and *Brucella melitensis* through cell SELEX. Iran J Vet Res 21:294–300.

**2. Direct diagnosis of S *Brucella* in domestic livestock**

**2.1. Valid for DSe/DSp assessment**

Comment. Only two studies. Tests were never implemented for routine use (i.e., not evaluated for robustness).

1. **Romero** C, Pardo ML, Grilló MJ, Díaz R, Blasco JM, López-Goñi I. 1995. Evaluation of PCR and indirect enzyme-linked immunosorbent assay on milk samples for diagnosis of brucellosis in dairy cattle. J Clin Microbiol 33:3198–3200. Negative controls: cows (n 37) from two brucellosis-free dairy herds. Positive controls: dairy cows (n 56) with field infection demonstrated by bacteriological culture (Farrell's and Thayer-Martin). PCR %DSe/%DSp (87,5/100). For milk iELISA, values were 98,2/100). Appropriate Dse/Dsp in milk. **COMMENTED IN TEXT.**

2. **Manterola** L, Tejero-Garces A, Ficapal A, Shopayeva G, Blasco JM, Marin CM, López- Goñi I: Evaluation of a PCR test for the diagnosis of *Brucella ovis* infection in semen samples from rams. Vet Microbiol 2003, 92:65–72. DSp 100% when testing semen samples from Brucella-free rams. Comparing the semen culture and PCR results from 192 semen samples tested showed a proportion of agreement of 0.91 between both tests. PCR has sensitivity similar (actually a bit less) to semen culture.

**2.2. Invalid for DSe, DSe and DSp, or DSp assessment**

General comments.

Many of these works use protocols not included in the 35 papers listed above.

Some works report "evidence" or "presence" of *Brucella* DNA. Many are "comparative" studies in undefined "control" groups, most often with no valid negative samples for DSp assessment. Some studies use imperfect bacteriology as a control for DSe and DSp. Most bacteriological studies are flawed (poor sensitivity because of insufficient inoculation, *B. melitensis* inhibitory selective medium, or non-selective media). Some report an ample number of PCR-positive results in seronegative cows, bulls, goats or sheep, or in many camels without clinical symptoms of any kind. Some report two or more species from the same animal, which has no precedents and is rather unlikely because a first infection triggers immunity against a second brucella infection. Some report contradictory results for serological tests, use tests not validated for the animal species or of low DSe (SAT). Specific comments are in italics.

3. Abdalla A, Hamid ME. 2012. Comparison of conventional and non-conventional techniques for the diagnosis of bovine brucellosis in Sudan. Trop Anim Health Prod 44:1151–1155. *Several poorly understood/validated serological tests. No appropriate positive controls; no appropriate negative controls; DSe/DSp assessment not possible*.

4. Akoko J, Pelle R, Kivali V, Schelling E, Shirima G, Machuka EM, Mathew C, Fèvre EM, Kyallo V, Falzon LC, Lukambagire AS, Halliday JEB, Bonfoh B, Kazwala R, Ouma C. 2020. Serological and molecular

evidence of *Brucella* species in the rapidly growing pig sector in Kenya. BMC Vet Res 16:133. *4 RBT+ and 16 RBT - analyzed; 4 RBT + PCR +; all 4 RBT + were cELISA - (!); RT-PCR+ in 2 RBT - pigs; No positive controls; no negative controls; DSe/DSp unknow*.

5. Al-Busadah KA, El-Bahr SM, Khalafalla AI. 2017. Serum biochemical profile and molecular detection of pathogens in semen of infertile male dromedary camels *Camelus dromedarius*. Anim Reprod Sci 180:58–65. *Commercial PCR kit claimed to be "validated" by the maker with no supporting information. No positive controls; no negative controls; DSe/DSp unknown*.

6. Al-Garadi MA, Khairani-Bejo S, Zunita Z, Omar AR. 2011. Detection of *Brucella melitensis* in blood samples collected from goats. J Anim Vet Adv 10:1437–1444. *RBT, CFT, Real time-PCR, and conventional PCR were compared in 288 goats of unknown status blood. No culture. (21 RBT - were RT-PCR +). No appropriate positive controls; no appropriate negative controls; DSe assessment not possible; /DSp assessment not possible*.

7. Al-Mariri A, Haj-Mahmoud N. 2010. Detection of *Brucella abortus* in Bovine Milk by Polymerase Chain Reaction. Acta Vet Brno 79:277–280. *Compares culture (OIE) and three different PCRs; one PCR identified 50/50 samples in filtrated milk; and 0/25 "noninfected" milk (defined how? By culture?). No negative controls. DSp assessment not possible*

8. Amin a S, Hamdy ME, Ibrahim a K. 2001. Detection of *Brucella melitensis* in semen using the polymerase chain reaction assay. Vet Microbiol 83:37–44. *Negative controls: semen (number not stated) from 5 brucellosis-free herds and flocks. Positive controls: semen from 65 bulls and 55 rams positive by RBT and variable SAT titers. PCR + 12/120; culture + 7/120. Poor bacteriology: unknown volume of semen samples cultured on Farrell (can inhibit B. melitensis). No appropriate negative controls; no appropriate positive controls; DSe/DSp assessment not possible.*

9. Amouei A, Sharif M, Sarvi S, Bagheri Nejad R, Aghayan SA, Hashemi-Soteh MB, Mizani A, Hosseini SA, Gholami S, Sadeghi A, Sarafrazi M, Daryani A. 2019. Aetiology of livestock fetal mortality in Mazandaran province, Iran. PeerJ 6:e5920. *No positive controls; no negative controls; DSe/DSp unknown.*

10. Beena V, Pawaiya RVS, Gururaj K, Singh DD, Mishra AK, Gangwar NK, Gupta VK, Singh R, Sharma AK, Karikalan M, Kumar A. 2017. Molecular etiopathology of naturally occurring reproductive diseases in female goats. Vet world 10:964–972. *PCR was done directly based on microscopic lesions; No appropriate positive controls; no negative controls; DSe assessment not possible; /DSp unknown.*

11. **C**apparelli R, Parlato M, Iannaccone M, Roperto S, Marabelli R, Roperto F, Iannelli D. 2009. Heterogeneous shedding of *Brucella abortus* in milk and its effect on the control of animal brucellosis. J Appl Microbiol 106:2041–2047. *Quantitative PCR of B. abortus positive milk samples gave comparable results to culture. Poor bacteriology: culture milk samples were enriched (also contaminants!) by overnight incubation at 37ºC in 10% $CO_2$. Cream and deposit were diluted ($10^{-1}$–$10^{-3}$ in $H_2O$ and 100 microL seeded on duplicate agar. There are no appropriate positive controls; no negative controls; DSe assessment not possible; /DSp unknown*.

12. Çiftci A, İça T, Savaşan S, Sareyyüpoğlu B, Akan M, Diker KS. 2017. Evaluation of PCR methods for detection of *Brucella* strains from culture and tissues. Trop Anim Health Prod 49:755–763. *Blood samples inoculated onto biphasic brain-heart infusion (BHI) agar; tissues etc. onto BHI agar with serum (not selective!). Despite poor bacteriology, PCR was not better. There are no appropriate positive controls; no negative controls; DSe assessment not possible; /DSp unknown.*

13. Costa LF, Pessoa MS, Guimarães LB, Faria AKS, Morão RP, Mol JP da S, Garcia LNN, Almeida AC, Gouveia AMG, Silva MX, Paixão TA, Santos RL. 2016. Serologic and molecular evidence of *Brucella ovis* infection in ovine and caprine flocks in the State of Minas Gerais, Brazil. BMC Res Notes 9:190. *A blind comparison (no culture) of ELISA, immunoprecipitation and PCR. There are no appropriate positive controls; no negative controls; DSe assessment not possible; /DSp unknown*.

14. Dos Santos LS, Sá JC, Dos Santos Ribeiro DL, Chaves NP, da Silva Mol JP, Santos RL, da Paixão TA, de Carvalho Neta AV. 2017. Detection of *Brucella* sp. infection through serological, microbiological, and molecular methods applied to buffaloes in Maranhão State, Brazil. Trop Anim Health Prod 49:675–679. *Several poorly understood/validated serological tests and PCR were used to "confirm" bacteriology; No appropriate positive controls; no negative controls; DSe assessment not possible; DSp unknown*.

15. Ebid M, El Mola A, Salib F. 2020. Seroprevalence of brucellosis in sheep and goats in the Arabian Gulf region. Vet World 13:1495– *Only animals RBT+/iELISA+ tested; PCR inferior to serology and "confirms" brucellosis. No appropriate of positive controls; no negative controls; DSe assessment not possible; /DSp unknown.*

16. Fatima S, Khan I, Nasir A, Younus M, Saqib M, Melzer F, Neubauer H, El-Adawy H. 2016. Serological, molecular detection and potential risk factors associated with camel brucellosis in Pakistan. Trop Anim Health Prod 48:1711–1718. *Several poorly understood/validated serological tests. No appropriate positive controls; no negative controls; DSe assessment not possible; DSp unknown.*

17. Fekete A, Bantle JA, Halling SM. 2015. Detection of *Brucella* by a polymerase chain reaction in bovine fetal and maternal tissues 79:5–9. *No culture. No positive controls; no negative controls; DSe/DSp unknown*.

18. Galluzzo P, Migliore S, Cascio S, Barreca S, Alfano M, Tagliarini A, Candela A, Piraino C, Galuppo L, Condorelli L, Hussein HA, Tittarelli M, Chiarenza G. 2021. Diagnostic Findings in a Confirmed Outbreak of *Brucella ovis* Infection in a Traditional Sheep Farm in Sicily South-Italy. Pathog 10, 1472. *Culture + in testes and epididymis of 7 animals (38.9%) / PCR+ in 13 (72.2%) testicles and epididymis. No negative controls; DSp assessment not possible*.

19. Gupta VK, Verma DK, Rout PK, Singh S V., Vihan VS. 2006. Polymerase chain reaction (PCR) for detection of *Brucella melitensis* in goat milk. Small Rumin Res 65:79–84. Polymerase chain reaction (PCR) for detection of Brucella melitensis in goat milk. *Goats (n 20) (defined as negative by culture) or a with history of abortion (n 54; 20 of culture-positive for B. melitensis); Incorrect criteria to define negative and positive controls. No appropriate positive controls; no negative controls; DSe assessment not possible/DSp assessment not possible*.

20. Hamdy MER, Amin a. S. 2002. Detection of *Brucella* species in the milk of infected cattle, sheep, goats and camels by PCR. Vet J 163:299–305. Negative controls were cows (n 50) from *Brucella*-free dairy herds. *Positive controls were 103 (52 cows, 21 sheep, 18 goats, 12 camels), from farms with a history of brucellosis (positive in at least one test [RBT, SAT or MRT]; tests of low sensitivity (SAT, MRT or, for camels, not validated). PCR compared to poor bacteriology (exceedingly low sensitivity in spiked samples; Farrell inhibitory for many B. melitensis strains). No appropriate positive controls; DSe assessment not possible*.

21. Hinić V, Brodard I, Thomann A, Holub M, Miserez R, Abril C. 2009. IS711-based real-time PCR assay as a tool for detection of *Brucella* spp. in wild boars and comparison with bacterial isolation and serology. BMC Vet Res 5:1–8. *Blind testing of RBT, iELISA, culture and PCR with no controls. No positive controls; no negative controls; DSe/DSp assessment not possible. Conclusion (IS711 real-time PCR assay is a specific and sensitive tool for detecting Brucella spp. infections in wild boars) unwarranted.*

22. Ilhan Z, Solmaz H, Aksakal A, Gulhan T, Ekin IH, Boynukara B. 2007. Comparison of PCR assay and bacteriological culture method for the detection of *Brucella melitensis* in stomach content samples of aborted sheep fetuses. Dtsch Tierarztl Wochenschr 114:460–464. *Serologically positive (n = 45) and negative (n = 117) slaughtered sheep. PCR + (41 ⁄45) from serologically positive sheep and (4 ⁄117) from serologically negative sheep. Of the 47 PCR + lymphoid tissue samples, 45 ⁄45 serologically positive sheep and 2 ⁄117 from serologically negative sheep. PCR assay detected a higher number of B. melitensis DNA from serologically positive and serologically negative PCR sheep than classical bacteriological culture. Very poor culture yield in seropositive animals. There are no appropriate positive controls; no appropriate negative controls; DSp assessment not possible; DSe assessment not possible*.

*23.* Junqueira-Junior DG, **R**osinha GMS, Carvalho CEG, Oliveira CE, Sanches CC. 2013. Detection of *Brucella* spp . DNA in the semen of seronegative bulls by polymerase chain reaction Transboundary and Emerging Diseases 60:376–377. *About 30% of 88 tested bulls PCR positive; all seronegative (no description of tests). Several poorly understood/validated serological tests. No culture. No positive controls; no negative controls; DSe/DSp unknown. COMMENTED IN TEXT*

24. Karthik K, Rathore R, Thomas P, Viswas KN, Agarwal RK, Rekha V, Jagapur R V, Dhama K. 2016. Rapid and visual loop mediated isothermal amplification (LAMP) test for the detection of *Brucella*

spp. and its applicability in epidemiology of bovine brucellosis. Vet Arh 86:35–47. *Comparative evaluation of LAMP, PCR, RBPT and SAT in 428 cattle whole blood samples. The specificity of LAMP was 100 %; sensitivity was 95.45 %, when compared to RBPT and 96.92 % when compared with SAT. No culture. An unexpected "correlation" between RBT and SAT questions the validity of serological controls. No appropriate negative controls; no appropriate positive controls; DSe/DSp assessment not possible*.

25. Khamesipour F, Doosti A, Rahimi E. 2015. Molecular study of Brucellosis in camels by the use of TaqMan® real-time PCR. Acta Microbiol Immunol Hung 62:409–421. *Several Brucella species detected in apparent healthy camels"/ no culture. DSe/DSp unknown. COMMENTED IN TEXT*

26. Leal-Klevezas DS, Martínez-Vázquez IO, García-Cantú J, López-Merino A, Martínez-Soriano JP. 2000. Use of polymerase chain reaction to detect *Brucella abortus* biovar 1 in infected goats. Vet Microbiol 75:91–97. *Number and origin of negative and positive controls (goats) not defined (same as Leal-Klevezas 1995a). No appropriate positive controls; no appropriate negative controls; DSe/DSp assessment not possible.*

27. Leyla G, Kadri G, Ümran O. 2003. Comparison of polymerase chain reaction and bacteriological culture for the diagnosis of sheep brucellosis using aborted fetus samples. Vet Microbiol 93:53–61. *126 aborted fetus samples of flocks of unknown brucellosis status (ewes not serologically tested). PCR and bacteriology (Farrell's medium) were positive in 38 and 39 samples, respectively. No culture-negative animal was PCR positive. Bacteriological method not optimized (Farrell is inhibitory for many B. melitensis strains). There are no appropriate positive controls; no appropriate negative controls; DSe assessment not possible; /DSp assessment not possible.*

28. Lindahl-Rajala, E.; Hoffman, T.; Fretin, D.; Godfroid, J.; Sattorov, N.; Boqvist, S.; Lundkvist, A.; Magnusson, U. Detection and characterization of *Brucella* spp. in bovine milk in small-scale urban and peri-urban farming in Tajikistan. PLoS Negl. Trop. Dis. 2017, 11, e0005367. *570 (568 for DNA testing) cows; serological tests: iELISA "confirmed "with cELISA; Brucella DNA was detected in the milk of all seropositive cows, but 8.3% of the seronegative cows (44 of 552) also showed the presence of Brucella DNA. Imperfect use of serological tests. No bacteriological controls. DSp assessment not possible.*

29. Lourencetti MPS, Souza MA, Ganda MR, Santos JP, Ferreira Júnior A, Miyashiro S, Lima AMC. 2018. High level of B19 strain detection in Brazilian cattle semen. Trop Anim Health Prod 50:433–439. *Negative controls were positive by PCR. COMMENTED IN TEXT*

30. Mahajan V, Banga HS, Filia G, Gupta MP, Gupta K. 2017. Comparison of diagnostic tests for the detection of bovine brucellosis in the natural cases of abortion. Iran J Vet Res 18:183–189. *Culture + 5/76; PCR + 27/108; No appropriate positive controls; no appropriate negative controls; DSe assessment not possible; /DSp assessment not possibl*e.

31. Manivannan et al. 2021. Molecular detection of brucellosis in dromedary camels of Qatar by real-time PCR technique. Comp Immunol Microbiol Infect Dis 78:101690. *All dromedary camels were healthy and clinically normal during the sample collection. Conclusion (the result "unveils that the most of the diseased camels are the noiseless transporters of Brucella") not warranted. No controls, either serological or bacteriological. DSe/DSp unknown. COMMENTED IN TEXT*

32. Marianelli C, Martucciello A, Tarantino M, Vecchio R, Iovane G, Galiero G. 2008. Evaluation of molecular methods for the detection of *Brucella* species in water buffalo milk. J Dairy Sci 91:3779–3786. *Of the 37 culture-positive samples, a total of 25 and 26 were positive by PCR and real-time PCR, respectively. Of the 16 culture-negative samples, 8 were positive by PCR and 9 by real-time PCR. PCR inferior to culture. No appropriate negative controls; DSp assessment not possible*

33. Moustacas Veria S, Silva TMA, Costa LF, Xavier MN, Carvalho C o dio A, Costa E rica A, Paix a o TA, Santos RL. 2013. Species-specific multiplex PCR for the diagnosis of *Brucella ovis*, *Actinobacillus seminis*, and *Histophilus somni* infection in rams. BMC Vet Res 9:51. *Experimentally infected rams; (those of B. ovis from Xavier et al 2010). DSe/DSp evaluation not applicable to natural infection*.

34. Nardi Júnior G, Megid J, Mathias LA, Paulin L, Vicente AF, Cortez A, Listoni FJP, Lara GHB, Motta RG, Chacur MGM, Monteiro FM, Ribeiro MG. 2017. Performance of microbiological, serological, molecular, and modified seminal plasma methods in the diagnosis of *Brucella abortus* in semen and serum of bovine bulls. Biologicals 48:6–9. *Several poorly understood/validated serological tests. PCR*

*+ in seronegative bulls. No appropriate positive controls; no appropriate negative controls; DSe assessment not possible; /DSp assessment not possible.*

35. Neha, Verma AK, Kumar A, Ahmed I. 2017. Comparative efficacy of serological diagnostic methods and evaluation of polymerase chain reaction for diagnosis of bovine brucellosis. Iran J Vet Res 18:279–281. *Poorly performed serological control tests/no culture. No appropriate positive controls; no appropriate negative controls; DSe assessment not possible; DSp assessment not possible.*

36. Ning P, Guo K, Xu L, Xu R, Zhang C, Cheng Y, Cui H, Liu W, Lv Q, Cao W, Zhang Y. 2012. Short communication: evaluation of *Brucella* infection of cows by PCR detection of *Brucella* DNA in raw milk. J Dairy Sci 95:4863–4867. *Polymerase chain reaction amplified Brucella DNA in 25 (45%)of the 55 SAT-positive cows. No culture. No appropriate positive controls; no appropriate negative controls; DSe assessment not possible. DSp unknown.*

37. Pérez-Sancho M, García-Seco T, Arrogante L, García N, Martínez I, Diez-Guerrier A, Perales A, Goyache J, Domínguez L, Alvarez J. 2013. Development and evaluation of an IS711-based loop mediated isothermal amplification method LAMP for detection of *Brucella* spp. on clinical samples. Res Vet Sci 95:489–494. *qPCR protocol applied to samples lung, spleen and liver from aborted foetuses and mammary lymph nodes and milk of 21 experimentally and aborted infected animals but not to samples from Brucella free animals; the conditions of the experimental infection were not described. DSe evaluation not applicable to natural infection; DSp unknown*.

38. Pilo C, Tedde MT, Orrù G, Addis G, Liciardi M. 2015. *Brucella suis* infection in domestic pigs in Sardinia, Italy. Epidemiol Infect 143:2170–2177. Atypical culture protocol not applied systematically. *No appropriate positive controls; no appropriate negative controls; DSe assessment not possible; DSp assessment not possible*.

39. Rahman SU, Zhu L, Cao L, Zhang Y, Chu X, Feng S, Li Y, Wu J, Wang X. 2019. Prevalence of Caprine brucellosis in Anhui province, China. Vet World 12:558–564. *180 samples: 7 RBT +, 8 SAT +; and 156 PCR +; i.e. , 148 PCR + correspond to serologically negative animals. COMMENTED IN TEXT*

40. Richtzenhain LJ e, Cortez A, Heinemann MB, Soares RM, Sakamoto SM, Vasconcellos SA, Higa ZMM, Scarcelli E, Genovez ME lide, Jose L, Bryan M, Martins R, Miyoshi S, Arruda S. 2002. A multiplex PCR for the detection of *Brucella* spp. and *Leptospira* spp. DNA from aborted bovine fetuses. Vet Microbiol 87:139–147. *Seven samples. Poor culture protocol: 0.1 ml of tissue suspensions and/or 0.1 ml of abomasal contents on selective medium; wrong interpretation "a higher rate of positive results was observed when PCR methods were employed, since they do not depend on the microbiological viability of the bacteria". No appropriate positive controls; no negative controls; DSe assessment not possible; DSp unknown.*

41. Saarangi LN, Polapally S, Rana SK, Bahekar VS, Surendra KSNL, Chandrasekhar Reddy RV, Raichur AS, Muthappa PN, Sharma GK. 2020. Development and laboratory validation of duplex real-time PCR for simultaneous detection of *Brucella* and bovine alphaherpesvirus from clinical specimens. Vet Ital 56. *DSe and DSp determined by screening 443 clinical specimens and comparing the results with non-described individual assays. No appropriate positive controls; no appropriate negative controls; DSp assessment not possible; DSe assessment not possible.*

42. Saini S, Gupta VK, Gururaj K, Singh DD, Pawaiya RVS, Gangwar NK, Mishra AK, Dwivedi D, Andani D, Kumar A, Goswami TK. 2017. Comparative diagnostic evaluation of OMP31 gene based TaqMan® real-time PCR assay with visual LAMP assay and indirect ELISA for caprine brucellosis. Trop Anim Health Prod 49:1253–1264. *PCRs compared with serological tests (SAT and iELISA) for DSe and DSp . Diagnostic sensitivities and specificities for TaqMan® real-time PCR vs. LAMP assays were 98 and 100% vs. 100 and 97.8%, respectively. Imperfect use of serological tests to define +/- controls. No appropriate positive controls; no appropriate negative controls; DSp assessment not possible; DSe assessment not possible.*

43. Sola MC**,** da Veiga Jardim EAG, de Freitas MR, de Mesquita AJ. 2014. Real-time PCR detection of *Brucella* spp. DNA in lesions and viscera of bovine carcasses. J Microbiol Methods 104:87–91. *A total of 276 samples with macroscopic changes suggestive (?) of brucellosis. No culture. No positive controls; no negative controls; DSe assessment not possible. DSp unknown.*

44. Tiwari A, Pal V, Afley P, Sharma DK, Bhatnagar CS, Bhardwaj B, Rai GP, Kumar S. 2014. Real-time PCR carried out on DNA extracted from serum or blood sample is not a good method for

surveillance of bovine brucellosis. Trop Anim Health Prod 46:1519–1522. *Randomly collected samples; RT-PCR shows no correlation with RBT or SAT; results of SAT vs RBT suggest that SAT + is autoagglutination. No appropriate positive controls; no appropriate negative controls; DSp assessment not possible; DSe assessment not possible*.

45. Tramuta C, Lacerenza D, Zoppi S, Goria M, Dondo A, Ferroglio E, Nebbia P, Rosati S. 2011. Development of a set of multiplex standard polymerase chain reaction assays for the identification of infectious agents from aborted bovine clinical samples. J Vet Diagn Invest 23:657–664. *Clinical samples from 50 aborted bovine fetuses without lesions suggestive of a specific infectious disease were randomly selected from different farms under the regional control program for bovine abortion. No culture. No appropriate positive controls; no negative controls; DSe assessment not possible. DSp unknown*.

46. Wareth G, Melzer F, Elschner MC, Neubauer H, Roesler U. 2014. Detection of *Brucella melitensis* in bovine milk and milk products from apparently healthy animals in Egypt by real-time PCR. J Infect Dev Ctries 8:1339–1343.*The study compares PCR and iELISA in an endemic area without a definition of individual status. ELISA cut-off for cattle according to the manufacturer. iELISA positive in 34 and RT-PCR in 17 milk samples. No appropriate positive controls; no appropriate negative controls; DSp assessment not possible; DSe assessment not possible.*

47. Wareth G, Melzer F, Tomaso H, Roesler U, Neubauer H. 2015. Detection of *Brucella abortus* DNA in aborted goats and sheep in Egypt by real - time PCR. BMC Res Notes 8:1–5. *Detection of both B. abortus and B. melitensis DNA in the same animal ("this study demonstrated that one host can be infected with two different species of Brucella at the same time"). No culture. There are no appropriate positive controls; no appropriate negative controls; DSp assessment not possible; DSe assessment not possible. COMMENTED IN TEXT*

48. Xavier MN**, S**ilva TMA, Costa EA, Paixão TA, Moustacas VS, Carvalho CA Junior, Sant'Anna FM, Robles CA, Gouveia AMG, Lage AP, Tsolis R, Tsolis R, Santos RL: Development and evaluation of a species-specific PCR assay for detection of *Brucella ovis* infection in rams. Vet Microbiol 2010, 145:158–164. *Experimentally infected or lyophilized semen from ELISA tested (variable results) rams; low sensitivity bacteriology; (PCR + in 28/40 of ELISA + semen samples and 16/23 of culture +); DSe evaluation not applicable to natural infection; PCR inferior to culture or serology, despite poor bacteriology in semen of naturally infected ram*s.

**2.4. Works that report PCR DSe inferior to (well performed) bacteriology in naturally infected animals but with no proper DSe controls**

49. Branscom LA, Cornish TE, Sondgeroth KS. 2019. Evaluation of serologic testing of rams in the management of *Brucella ovis* in a domestic sheep flock. J Vet Diagn Invest 31:86–89. *Direct culture identified B. ovis in a higher proportion of rams for every tissue compared to PCR, except for the right bulbourethral gland, in which culture and PCR had similar results, and in inguinal lymph medial iliac lymph nodes (both culture and PCR negative). No appropriate negative controls; DSp assessment not possible.*

*50.* Chisi SL, Schmidt T, Akol GW, Van Heerden H. 2017. Use of *Brucella abortus* species-specific polymerase chain reaction assay for the diagnosis of bovine brucellosis. J S Afr Vet Assoc 88:e1-e3. 48 samples from farms with an abortion history. Of these, 10 culture + ( OIE method and 7 PCR +. *The remaining 38 samples were also tested with the BaSS PCR: only 2 culture-negative abomasal fluid samples amplified a 350 bp fragment unique to B. abortus RB51. No appropriate negative controls; DSp assessment not possible.*

51. Saytekin AM, Ak S. 2018. Direct diagnosis of *Brucella* species through multiplex PCR formed by a new method. J Microbiol Methods 154:86–94. *Culture method was set as the standard method for DSe and DSp. Fetus n=166, DSe and DSp values for modified Mayer-Scholl m-PCR method were 94.11% and 98.76%; for the modified Bcsp31 PCR were 95.29% and 98.76%. Organ samples n=326, DSe, and DSp for the modified Mayer-Scholl m-PCR method were 85.38% and 98.06%, and for the modified Bcsp31 PCR, 83.62% and 98.06%. DSe of PCR inferior to culture. No appropriate negative controls; DSp assessment not possible.*

52. Buyukcangaz E, Sen A, Carli KT, Kahya S. 2011. Comparison of direct culture versus PCR for the detection of *Brucella* in aborted fetuses of cattle and sheep in Turkey. Vet Rec 168:430. *Organ homogenates from 38 aborted fetuses of cattle and 56 aborted fetuses of sheep (commercial PCR*

*kit). Culture 30 (8 from cattle; 22 from sheep)/94. PCR +, 29 (8 from cattle, 21 from sheep) / 94. Compared to culture, DSe/DSp were 83 and 94 %. Wrong negative control (bacteriology). DSe of PCR inferior to culture; No appropriate negative controls; DSp assessment not possible.*

53. Vejarano MP, Matrone M, Keid LB, Rocha VCM, Ikuta CY, Rodriguez CAR, Salgado VR, Ferreira F, Dias RA, Telles EO, Ferreira Neto JS. 2013. Evaluation of four DNA extraction protocols for *Brucella abortus* detection by PCR in tissues from experimentally infected cows with the 2308 strain. Vector Borne Zoonotic Dis 13:237–242. *DSe evaluation does not apply to natural infections. All of the DNA extraction protocols resulted in false-negative results for PCR. PCR inferior to culture.*

54. O'Leary, S., Sheahan, M. & Sweeney, T., 2006, '*Brucella abortus* detection by PCR assay in blood, milk and lymph tissue of serologically positive cows', Research in Veterinary Science 81, 170–176. https://doi.org/10.1016/j.rvsc.2005.12.001. *Study of seropositiv animals aimed to evaluate PCR to ascertain the Brucella species instead of bacteriology. Well-performed bacteriology of seropositive cattle. There was no difference between PCR and bacteriological detection methods. Conclusion: "It is unlikely that conventional or real-time PCR will supersede current diagnostic methods for detection of B. abortus in clinical samples." Authors point out that the stage of infection can affect detection. Experimental design precludes concluding DSp; not true Dsp/Dse assessment.*

**2.5. Bayesian studies**

General comment: one study; unclear rationale for choosing the model (conditional dependence between two of the tests and independence of the third test). Priors were taken from unrelated studies (PCR of whole blood and paraffin-embedded tissues or diagnosis of canine brucellosis or studies with no appropriate positive/negative controls)

*55.* Nyarku R, Hassim A, Jonker A, Quan M. 2020. Development of a Genus-Specific *Brucella* Real-Time PCR Assay Targeting the 16S-23S rDNA Internal Transcribed Spacer from Different Specimen Types. Vet Sci 7. *Flawed Bayesian study (see below). COMMENTED IN TEXT*

DSe/DSp assessement: *A blind study of random positive and negative samples (no description of how they were classified as positive and negative)*. "Diagnostic sensitivity and specificity of the PCR assay were estimated in the absence of a gold standard assay, by using a three-test one-population Bayesian latent class model that allowed for conditional dependence between two of the tests and independence of the third test [31]. Modes were obtained from published references". *These were:*

Kattar, M.M.; Zalloua, P.A.; Araj, G.F.; Samaha-Kfoury, J.; Shbaklo, H.; Kanj, S.S.; Khalife, S.; Deeb, M. Development and evaluation of real-time polymerase chain reaction assays on whole blood and paraffin-embedded tissues for rapid diagnosis of human brucellosis. Diagn. Microbiol. Infect. Dis. 2007, 59, 23–32. [CrossRef]

Keid, L.B.; Soares, R.M.; Vieira, N.R.; Megid, J.; Salgado, V.R.; Vasconcellos, S.A.; da Costa, M.; Gregori, F.; Richtzenhain, L.J. Diagnosis of canine brucellosis: Comparison between serological and microbiological tests and a PCR based on primers to 16S-23S rDNA interspacer. Vet. Res. Commun. 2007, 31, 951–965.

Richtzenhain, L.J.; Cortez, A.; Heinemann, M.B.; Soares, R.M.; Sakamoto, S.M.; Vasconcellos, S.A.; Higa, Z.M.; Scarcelli, E.; Genovez, M.E. A multiplex PCR for the detection of Brucella spp. and Leptospira spp. DNA from aborted bovine fetuses. Vet. Microbiol. 2002, 87, 139–147 (*See above; this study had no appropriate positive controls; no negative controls; DSe assessment not possible; DSp unknown*)

Güler, L.; Kadri, G.; Umran, O. (actually Leyla et al. see above) Comparison of polymerase chain reaction and bacteriological culture for the diagnosis of sheep brucellosis using aborted fetus samples. Vet. Microbiol. 2003, 93, 53–61. (See above; *No appropriate positive controls; no appropriate negative controls*;

For multiple references, the average of the modes was used (Table 2). For the sensitivity of the 16S-23S rDNA ITS PCR assay, the references reported on the diagnostic sensitivity in blood samples. For the diagnostic sensitivity in aborted material, the prior for this parameter was adjusted upwards. No reference could be obtained for "prevalence", the proportion of tested aborted material positive for Brucella spp. submitted to a diagnostic laboratory in South Africa and expert opinion (Dr. A. Jonker, DVTD, UP) was obtained.
